# Antithrombotic Therapy in Patients with Acute Coronary Syndrome in the Intermountain Heart Collaborative Study

**DOI:** 10.1155/2015/270508

**Published:** 2015-01-08

**Authors:** Stacey Knight, Winslow Klaskala, Scott C. Woller, Benjamin D. Horne, T. Jared Bunch, Viet T. Le, Roger M. Mills, Joseph B. Muhlestein

**Affiliations:** ^1^Intermountain Medical Center, Intermountain Heart Institute, 5121 S. Cottonwood Street, Murray, UT 84107, USA; ^2^University of Utah School of Medicine, Salt Lake City, UT 84108, USA; ^3^Janssen Research and Development, Raritan, NJ 08869, USA

## Abstract

*Objective*. To determine factors associated with single antiplatelet (SAP) or dual antiplatelet (DAP) therapy and anticoagulants (AC) use in hospital and after discharge among patients with acute coronary syndrome (ACS). *Methods*. We evaluated 5,294 ACS patients in the Intermountain Heart Collaborative Study from 2004 to 2009. Multivariable logistic regressions were used to determine predictors of AC or AP use. *Results*. In hospital, 99% received an AC, 79% DAP, and 19% SAP; 78% had DAP + AC. Coronary stents were the strongest predictors of DAP use in hospital compared to SAP (*P* < 0.001). After discharge, 77% received DAP, 20% SAP, and 9% AC; 5% had DAP + AC. DAP compared to SAP was less likely for patients on AC (odds ratio [OR] = 0.30, *P* < 0.0001) after discharge. Placement of a stent increased the likelihood of DAP (bare metal: OR = 54.8, *P* < 0.0001; drug eluting: OR = 59.4, *P* < 0.0001). 923 had atrial fibrillation and 337 had a history of venous thromboembolism; these patients had increased use of AC (29% and 40%, resp.). *Conclusion*. While in-hospital use of AC was nearly universal, postdischarge AC use was rare. Concern for providing the best antithrombotic therapy, while maintaining an acceptable bleeding risk, may explain the selection decisions.

## 1. Introduction

Acute coronary syndrome (ACS) is a manifestation of unstable atherothrombotic coronary artery disease requiring prompt intervention. ACS is characterized by intracoronary thrombosis involving platelet activation and fibrin formation. This process occurs acutely but may also persist for months to years following initial ACS [1]. In the absence of both immediate and long-term attempts to mitigate thrombosis with a variety of antiplatelet (AP) and anticoagulant (AC) agents, recurrent ACS is common and may result in death or significant disability [2, 3]. Multiple trials of ACS demonstrate the benefit of AP agents [2, 4–7]. While the cornerstone of secondary prevention of ACS is historically AP therapy, some studies suggest possible benefit attributable to complementary AC therapy (with others showing no benefit) [8–11]. However, this benefit might not compensate for the risk of a major bleed [9–12].

Diseases that necessitate AC therapy, including atrial fibrillation (AF) and venous thromboembolism (VTE), often coexist with ACS. These comorbidities significantly increase the risk of adverse events in ACS patients. Patients with ACS and AF have a higher rate of in-hospital stroke and major bleeding and a more than threefold increase in rates of in-hospital mortality compared to ACS without AF [13]. Likewise, ACS patients with coexistent deep venous thrombosis or pulmonary embolus have an increased risk of death compared with ACS patients without these diagnoses [14–16]. Among patients with VTE anticoagulation remains the treatment of choice [17–19]. Therefore, in stented ACS patients with coexistent thrombotic processes, strict adherence to clinical guidelines for management of these, often comorbid, diseases results in the impetus to use both dual antiplatelet (DAP) and AC therapy [20–22]. Yet the quality of evidence leading to guideline recommendations among patients that require DAP and AC therapy is low given that virtually all ACS antithrombotic clinical trials have excluded patients with coexistent thrombotic diseases. Hence, very little objective evidence supports the DAP plus AC (“triple therapy”) strategy [23].

Clinical decision-making regarding these complex patients requires weighing the benefit attributable to multiple antithrombotic (AT) agents against the risk of bleeding. How clinicians utilize existing guidelines for each coexistent thrombotic disease process as well as what AT strategy they choose has not been well described. The aim of this study is to describe the patterns of both in-hospital and postdischarge AT therapy for patients presenting with ACS in the presence or absence of other coexistent thrombotic disease processes.

## 2. Methods

This study was approved by the Intermountain Healthcare institutional review board. Investigations were in accordance with the Declaration of Helsinki. A prospective observational cohort study design using electronic data was employed.

### 2.1. Study Population

Study subjects were identified from all persons registered in the Intermountain Heart Collaborative Study (IHCS) with a diagnosis of ACS between 2004 and 2009 who survived to discharge and had documentation of in-hospital and postdischarge medications (*n* = 5294). IHCS enrolls patients admitted to Intermountain catheterization labs (Intermountain Medical Center: Salt Lake City, UT; LDS Hospital: Salt Lake City, UT; and McKay Dee Hospital, Ogden, UT) [24]. ACS was defined by the presence of the following International Classification of Disease 9th version (ICD9) codes: 410.x (acute myocardial infarction including STEMI and NSTEMI) and 411.x (unstable angina). ACS diagnosis was verified on a subset of the patients using manual chart review. The identified cohort was divided into six groups according to the AT therapy received during hospitalization for the incident ACS event and then upon discharge. The AT variable was categorized based on AP (e.g., aspirin, clopidogrel, and other AP such as prasugrel and ticlopidine) use as no AP, single AP (SAP), or DAP. Patients were further subdivided into AC therapy (yes/no) which included warfarin and other anticoagulants (e.g., heparin, low molecular weight heparin, bivalirudin, and fondaparinux).

Other data derived electronically include patient demographics, cardiac risk factors (e.g., hypertension, diabetes, hyperlipidemia, smoking status, and family history of heart disease), clinical presentation, diagnosis, percutaneous coronary intervention, coronary artery bypass grafting, and medications received (both in-hospital and postdischarge). Comorbidities were defined based on ICD9 codes (Supplementary Table 1, available online at http://dx.doi.org/10.1155/2015/270508) at prior visits in the Intermountain Healthcare System or at the time of the index hospitalization, except for hypertension and hyperlipidemia, which were both defined using physician notes. Major bleeding was defined as symptomatic bleeding (using ICD9 codes) in a critical area or organ (such as intracranial bleeding, intraspinal intraocular, retroperitoneal, intra-articular or pericardial, or intramuscular with compartment syndrome) and/or leading to transfusion of two or more units of blood. Patient risk scores for thromboembolic stroke were calculated using the CHADS_2_ score. The CHADS_2_ score (congestive heart failure; hypertension; age; diabetes; previous ischemic stroke) is a simple and commonly used risk assessment tool for predicting stroke risk in patients with nonvalvular AF. Patients were assigned 2 points for a history of stroke or TIA, and 1 point each was assigned for a history of heart failure, hypertension, diabetes, or age >75 years [25].

### 2.2. Statistical Analysis

The purpose of the analyses is to explore the factors, both individually and as a group, associated with in-hospital and postdischarge AC and AP therapies for ACS patients. Chi-square tests and logistic regression were used for the univariate analyses of factors (those variables listed in Tables 2 and 3) associated with AP and AC use. Significant factors (*P* ≤ 0.1) in the univariate analyses were incorporated into multivariate logistic regression models used to explore the overall relationships between factors and AT use. For AC use analyses, we chose a priori to stratify by SAP and DAP to allow for the prediction of AC use given the prescribed AP regimen as this was felt to be the most beneficial for clinicians. SAS 9.2 (SAS Institute, Cary North Carolina) was used for all the analyses. Proc Logistic was used for the multivariate logistic regression and 95% confidence intervals are reported for the odds ratios (OR) with a significance level set at *P* ≤ 0.05.

## 3. Results

### 3.1. Patient Characteristics and AT Prescription Practices

The majority of enrolled ACS patients received DAP in-hospital (78.7%) and postdischarge (77.3%) (Table 1). DAP consisted mainly of aspirin and clopidogrel for both in-hospital (98.4%) and postdischarge (99.5%) (Supplementary Table 2). Almost all (99.4%) of the patients received at least one form of in-hospital AC therapy (87% heparin, 18% low molecular weight heparin, 12% warfarin, and 7% other); however, only 9.0% received AC postdischarge (100% warfarin) (Table 1). At discharge, patients not on an AC (*n* = 4819) were more likely to have DAP (79.2%), while those patients on AC (*n* = 475) were not likely to be on DAP (57.5%). Triple AT therapy was used in-hospital for the majority (78.5%) of the ACS patients; however, only 5.2% had triple therapy after discharge. Major bleeding occurred in 17 patients during hospitalization. All of these had AC use and 50% were concomitantly on DAP. The patient characteristics for in-hospital AT therapy are shown in Table 2.

The patient characteristics for postdischarge AT therapy are shown in Table 3. In the 923 AF and ACS patients, 29.0% received AC after discharge and of 337 VTE and ACS patients, 39.8% received AC after discharge. About a third (32.0%) of the VTE events identified occurred during hospitalization and thus this could drive the choice of both in-hospital and postdischarge AT therapy.  Table 4 contains the postdischarge AT therapy for specific procedures and AC indications (i.e., AF with CHADS_2_ score ≥2 or VTE during hospitalization). The majority (75%–95%) of patients that had no indication of AC use received appropriate postdischarge AT therapy, regardless of procedures. If the patient had an indication for AC use, only about a quarter of the AF with CHADS_2_ score ≥2 patients and a third to a half of the patients that had a VTE during hospitalization received postdischarge AC therapy.

### 3.2. DAP versus SAP Use

Coronary stents, both bare metal and drug eluting, were the strongest predictors of an increase in the likelihood of DAP as compared with SAP in-hospital (bare metal: OR = 24.0, *P* < 0.001; drug eluting: OR = 17.3, *P* < 0.0001), while CABG decreased the likelihood of DAP (OR = 0.11, *P* < 0.0001) (Supplementary Figure 1). Other factors in the multivariable analysis associated with increased likelihood of hospital DAP versus SAP were multi- or single vessel CAD disease compared to no vessel disease (single: OR = 3.1, *P* < 0.0001; multivessel OR = 4.1, *P* < 0.0001), prior MI (OR = 1.5, *P* = 0.004), hyperlipidemia (OR = 1.3, *P* = 0.025), and increase in length of stay per day (OR = 1.02, *P* = 0.040). VTE (OR = 0.61, *P* = 0.023) and persistent AF (OR = 0.59, *P* = 0.035) were associated with a decreased likelihood of hospital DAP.

**Figure 1:**
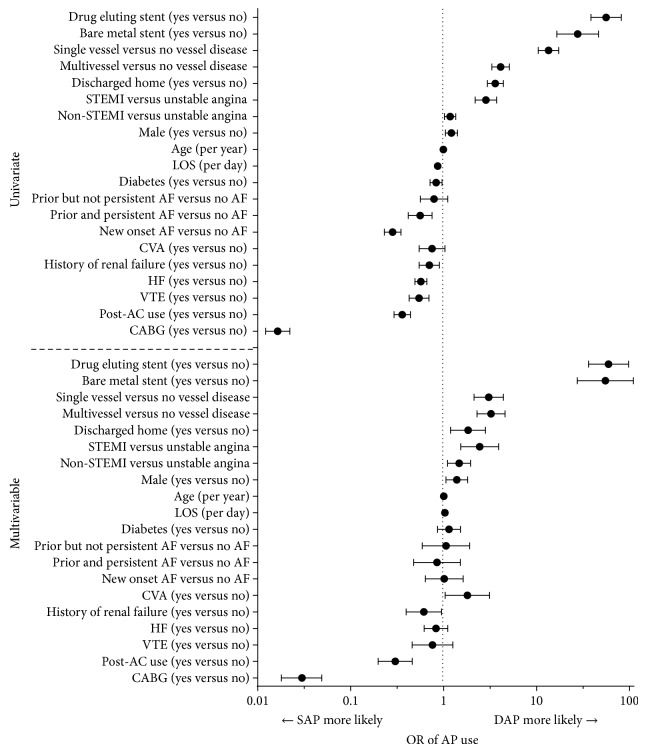
Univariate and multivariable logistic regressions for postdischarge SAP versus DAP use. DAP: dual antiplatelet; SAP: single antiplatelet; OR: odds ratio; STEMI: ST elevated myocardial infarction; LOS: length of stay; AF: atrial fibrillation; CVA: cerebral vascular accident; HF: heart failure; VTE: vascular thrombus event; A: anticoagulant; CABG: coronary artery bypass graph.

The postdischarge univariate and multivariate analysis of SAP versus DAP are shown in Figure 1. AC use occurred less frequently among patients receiving DAP compared to SAP even after adjusting for other factors (OR = 0.30, *P* < 0.0001). The placement of coronary stents increased the likelihood of DAP therapy (bare metal: OR = 54.8, *P* < 0.0001; drug eluting: OR = 59.4, *P* < 0.0001), while CABG decreased the likelihood of DAP (OR = 0.03, *P* < 0.0001). Other factors in the multivariable analysis associated with an increased likelihood of postdischarge DAP were single and multivessel disease, MI (compared to unstable angina), discharge to home, CVA, increased length of stay at the index hospitalization, and male gender. Renal failure was associated with decreased DAP use at discharge.

### 3.3. AC Use

**Figure 2:**
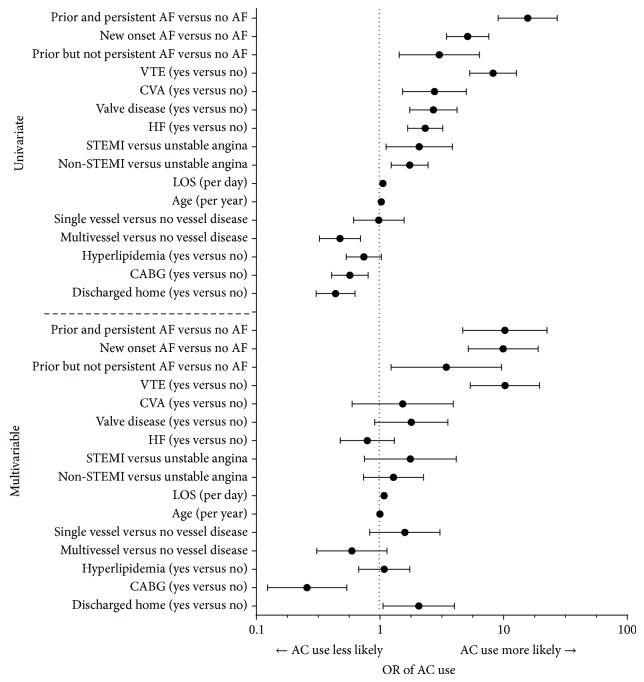
Univariate and multivariable logistic regressions for postdischarge AC use for SAP. AC: anticoagulant; SAP: single antiplatelet; OR: odds ratio; STEMI: ST elevated myocardial infarction; LOS: length of stay; AF: atrial fibrillation; CVA: cerebral vascular accident; HF: heart failure; VTE: vascular thrombus event; CABG: coronary artery bypass graph; CVA: cerebral vascular accident.

As almost all patients (99.4%) received AC therapy in-hospital, analyses exploring the factors associated with in-hospital AC therapy were not done. The univariate and multivariable analyses for postdischarge AC use by patients on SAP after discharge are shown in Figure 2. In the multivariable analysis, history of VTE (OR = 10.3, *P* < 0.0001), AF status (new onset: OR = 9.9, *P* < 0.0001, and persistent: OR = 10.2, *P* < 0.0001), increasing hospital length of stay (OR = 1.1, *P* < 0.0001), and being discharged to home (OR = 2.1, *P* = 0.033) were all associated with an increased likelihood of AC use. We used discharge to home as a marker for frailty as it was found to be significantly negatively correlated (*P* < 0.0001) with prior diagnoses for any of the following: past medical history of treated falls, dementia, delirium, and cognitive impairment. CABG was associated with a decreased likelihood of AC use (OR = 0.26, *P* = 0.0004).

**Figure 3:**
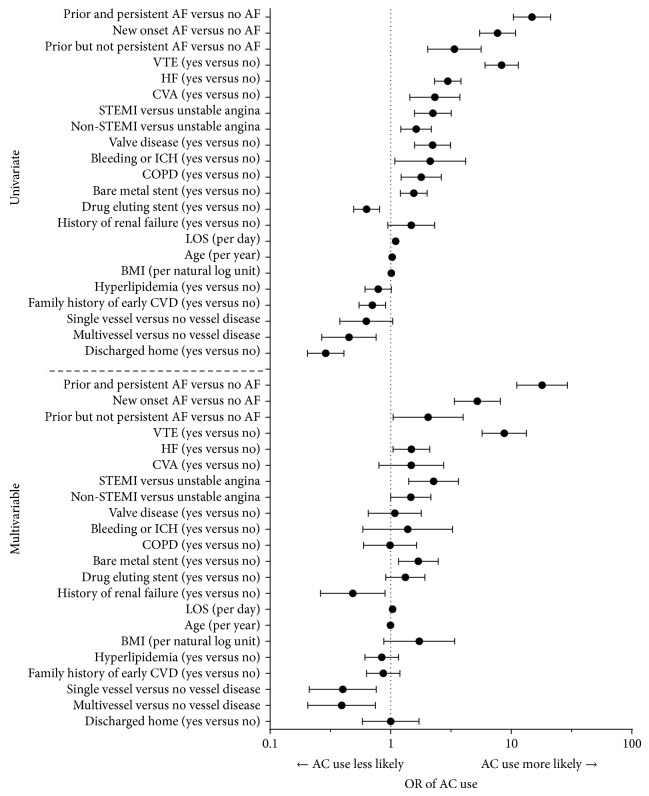
Univariate and multivariable logistic regressions for postdischarge AC use for DAP. AC: anticoagulant; DAP: dual antiplatelet; OR: odds ratio; STEMI: ST elevated myocardial infarction; LOS: length of stay; AF: atrial fibrillation; CVA: cerebral vascular accident; HF: heart failure; VTE: vascular thrombus event; CABG: coronary artery bypass graph; CVA: cerebral vascular accident; ICH: intracranial hemorrhage; COPD: chronic obstructive pulmonary disease; CVD: cardiovascular disease.

The univariate and multivariable analyses for postdischarge AC use by patients on DAP at discharge are shown in Figure 3. Similar to the results for patients on SAP, in the multivariable analysis for patients on DAP, history of VTE (OR = 8.7, *P* < 0.0001) and AF status (new onset: OR = 5.2, *P* < 0.0001, and persistent: OR = 18.0, *P* < 0.0001) were associated with an increased likelihood of AC use. Additionally, the presentation of MI compared to unstable angina (STEMI: OR = 2.3, *P* = 0.0007; non-STEMI: OR = 1.5, *P* = 0.049), the use of a bare metal stent (OR = 1.7, *P* = 0.006), heart failure (OR = 1.5, *P* = 0.028), and hospital length of stay (OR = 1.03, *P* = 0.027) were associated with greater AC use. A history of renal failure was associated with a decreased likelihood of AC use (OR = 0.49, *P* = 0.021). Single and multivessel disease compared to no vessel disease were associated with a decreased likelihood of AC use (single: OR = 0.39, *P* = 0.005; multivessel: OR = 0.40, *P* = 0.005).

## 4. Discussion

These data describe the AT prescribing patterns of physicians caring for patients who were admitted with ACS, in particular, ACS patients with coexisting illnesses such as AF or VTE. We identified several interesting prescribing practice trends.

First, although a majority (79%) of patients presenting with ACS received triple AT therapy during the time of hospitalization, only a small minority (5%) received triple AT after discharge. Even among the patients with an indication for long-term anticoagulation, only about a quarter were discharged with appropriate antiplatelet and anticoagulation therapies. This implies that even though strict adherence to the multiple management guidelines associated with treatment of coexistent thrombotic diseases may indicate prescription of both AC and DAP therapy at discharge, providers in general are reluctant to do so. Perhaps this is attributable to the higher risk of bleeding associated with triple AT therapy. In a study of patients with AF who underwent percutaneous coronary stenting, the risk of late major bleed was more than five times higher among those discharged with triple AT therapy [26]. Additionally, in a meta-analysis of 1,996 participants on chronic long-term AC therapy who underwent coronary artery stenting, the risk of major bleeding was doubled among patients discharged with triple therapy [27]. However, despite the higher reported risk of bleeding with triple therapy, the same study also reported a 40% reduction in major adverse cardiovascular events and a 41% reduction in all-cause mortality among those patients who received triple therapy. Recent guidelines additionally recommend limited (one month) triple therapy for select patients (e.g., patients with anterior MI and LV thrombus, or at high risk for LV thrombus—ejection fraction <40% plus and anteroapical wall motion abnormality—who undergo BMS/DES placement) [17]. This was a weak (grade of 2C) recommendation based on observational and case-control studies, not randomized clinical trials. Although triple therapy may reduce the risk of ischemic complications in these patients at the cost of increased bleeding, our study confirms that, in general, providers perhaps disproportionately restrict their use of long-term triple AT therapy.

Second, AF and VTE were the most common diagnoses associated with discharge AC therapy. Both of these conditions have strong evidence-based indications for chronic AC. It is rational that providers would prioritize AC for AF and VTE patients with ACS. In circumstances where DAP therapy is imperative, such as when a drug eluting stent has been deployed, temporary interruption of chronic AC may occur. Limited data in this circumstance suggest clinical benefit for DAP therapy as reported in the “Atrial Fibrillation Clopidogrel Trial with Irbesartan for Prevention of Vascular Events (ACTIVE) A” trial [28]. However after an initial treatment duration of 3 months for acute VTE, continuation of anticoagulation for secondary prevention is recommended based upon risk of recurrence and individual patient values and preferences [18, 19]. It is possible that, in some cases studied, the ACS event introduced an opportunity to reassess overall risk and benefit leading to the deliberate cessation of AC therapy.

Third, most patients (77%) received DAP therapy upon discharge. However when AC was prescribed in addition to AP therapy, the use of DAP therapy was less frequent (57%) than when no AC was given (79%). In general, when a single AP agent was used, aspirin was chosen over clopidogrel (95.3% versus 4.5%). The reasons for this are not clear, and the choice may reflect economics or tradition. However, the limited available evidence suggests that AC plus SAP therapy with a P2Y12 inhibitor (clopidogrel) rather than aspirin may be preferable. In the “What is the optimal antiplatelet and anticoagulant therapy in patients with oral anticoagulation and coronary stenting (WOEST)” trial, patients with an indication for chronic AC undergoing coronary stent procedures were randomized to triple AT therapy or dual therapy with warfarin plus clopidogrel, excluding the use of aspirin [29]. During one year of follow-up, patients randomized to warfarin/clopidogrel experienced both a 64% reduction in bleeding complications and a 40% reduction in ischemic complications as compared to those randomized to triple AT.

Fourth, we observed increased odds of AC prescription at discharge among patients receiving bare metal stents during PCI. The obvious advantage of such an approach is the fact that the duration of required DAP therapy is significantly shorter when a bare metal rather than a drug eluting stent is used. Indeed this advantage has reached to the level of inclusion in national guidelines. In the 2011 ACC/AHA guidelines for percutaneous coronary intervention concern over the use of drug eluting stents in patients in which chronic oral AC is indicated is expressly stated [30]. The findings in this study suggest that these recommendations have entered into practice.

The optimal strategy for AT therapy, employing a combination of AP agents and anticoagulation, is uncertain. For clinicians, decision-making often reflects the perceived risk of bleeding and thrombosis associated with multiple comorbidities both during and following hospitalization. Coexisting diseases that may have independent indications for AC or AP therapy are common, affecting up to 25% of all ACS patients. With ever increasing options for AT treatment of patients with ACS, the challenge for providers to choose the best strategy for each individual will continue to increase. For example, in this retrospective study virtually all patients receiving chronic oral AC therapy received warfarin; now, four other novel oral anticoagulants are available [31]. Two have been tested as an adjunct to dual AP therapy in the setting of ACS without any coexisting thrombotic processes requiring chronic AC [32, 33].

There are limitations with this study. This was a retrospective observational study and as such there are inherent limitations with the data. These include lack of detailed information about the reasons surrounding the prescribing decision. These decisions could include a patient's refusal of AC treatment or contraindications for AC treatment. We believe such situations are rare and that most prescribing decisions are based on the physician's choice. Observational data is also prone to incomplete datasets. To minimize this for the key variables of ACS and AT treatment, we did perform manual chart review on a subset of the patients. The missing data was minimal and there did not appear to be any systematic bias that would impact our conclusions. Finally, this study was conducted in one integrated healthcare system. While this may impact generalizability to other settings, the findings do seem to be consistent with the reported literature. Despite these limitations, this study provides a comprehensive examination of physician AT prescribing practices in a large ACS population.

## 5. Conclusions

This study describes AT “real-world” management strategies for patients presenting with ACS. In-hospital, virtually all ACS patients were anticoagulated, and 80% also received DAP therapy. After discharge, AC was continued for a minority of ACS patients with coexisting AF or VTE, well-established indications for AC. When AC was used, DAP therapy was used significantly less often. These data suggest that the clinician's concern for providing optimal AT therapy also included achieving an acceptable overall risk for bleeding. In general, the decisions regarding AT therapy management in this study appeared to be made rationally. The absence of evidence-based data from adequate randomized clinical trials of AT options for ACS patients with coexisting AF or VTE likely explains much of the practice variability identified. Newly available AT agents will make the decision process even more complicated in the future.

## Supplementary Material

The supplementary materials include two tables and one figure. The first table contains the ICD-9 codes that were used to define each of the comorbidities. The second table contains the actual medications that make up each category of the single (SAP) or dual (DAP) antiplatelet and anticoagulants (AC). The figure contains the univariate and multivariable logistic regression for odd ratios for factors associated with in-hospital SAP vs DAP use.

## Figures and Tables

**Table 1:** Antiplatelet (AP) and anticoagulant (AC) use both in-hospital and postdischarge.

	In-hospital	Postdischarge
	*n* = 5294	*n* = 5294
DAP	4165 (78.7%)	4091 (77.3%)
SAP	1012 (19.1%)	1077 (20.3%)
No AP	117 (2.2%)	126 (2.4%)
AC	5264 (99.4%)	475 (9.0%)
No AC	30 (0.6%)	4819 (91.0%)
Triple therapy (DAP + AC)	4155 (78.5%)	273 (5.2%)

Note: DAP: dual antiplatelet; SAP: single antiplatelet.

**Table 2:** Patient characteristics by in-hospital AT therapy.

	No Antiplatelet		Single antiplatelet		Dual antiplatelet	
	No AC	AC	No AC	AC	No AC	AC
	*n* = 8	*n* = 109	*n* = 12	*n* = 1000	*n* = 10	*n* = 4155
Age (mean ± std)	63.8 ± 13.48	63.1 ± 15.25	68.4 ± 16.87	64.7 ± 12.50	66.9 ± 10.93	64.1 ± 12.25
Male	37.5%	67.9%	58.3%	68.4%	80.0%	74.2%
Caucasian	87.5%	91.7%	91.7%	88.2%	100.0%	89.7%
Body Mass Index (mean ± std)	29.5 ± 4.18	28.9 ± 6.29	27.4 ± 5.93	30.0 ± 10.04	32.2 ± 7.44	29.7 ± 7.55
Family history of early CVD	50.0%	36.7%	33.3%	39.7%	40.0%	45.3%
Smoking history	25.0%	13.8%	16.7%	15.2%	10.0%	16.4%
Prior MI	25.0%	7.3%	0.0%	13.8%	0.0%	17.5%
Comorbidities						
COPD	0.0%	8.3%	25.0%	7.6%	0.0%	7.5%
CVA	0.0%	5.5%	8.3%	5.1%	0.0%	3.8%
Diabetes	12.5%	21.1%	16.7%	26.5%	30.0%	25.9%
Heart failure	25.0%	25.7%	33.3%	31.8%	10.0%	22.6%
Hypercoagulability	0.0%	0.9%	0.0%	0.4%	0.0%	0.1%
Hyperlipidemia	37.5%	57.8%	58.3%	60.3%	50.0%	66.2%
Hypertension	50.0%	65.1%	75.0%	62.6%	90.0%	66.0%
Major bleeding or ICH	0.0%	2.8%	8.3%	2.8%	0.0%	1.9%
Renal failure	0.0%	7.3%	0.0%	8.2%	20.0%	6.4%
Valve disease	0.0%	11.9%	0.0%	9.2%	10.0%	9.0%
VTE	0.0%	9.2%	0.0%	9.1%	10.0%	5.7%
AF						
No AF	75.0%	81.7%	58.3%	72.5%	90.0%	85.1%
Prior but not current	12.5%	6.4%	16.7%	3.8%	0.0%	4.2%
Prior and current	0.0%	8.3%	0.0%	6.6%	10.0%	4.2%
New onset	12.5%	3.7%	25.0%	17.1%	0.0%	6.5%
Charlson Comorbidity Index (mean ± std)	5.6 ± 3.74	6.0 ± 3.72	8.2 ± 5.02	6.5 ± 4.05	6.6 ± 5.60	5.9 ± 3.82
Mechanical value	0.0%	0.0%	0.0%	0.5%	0.0%	0.2%
Presentation						
Unstable angina	25.0%	65.1%	41.7%	44.2%	20.0%	40.0%
STEMI	0.0%	9.2%	16.7%	8.2%	30.0%	14.6%
Non-STEMI	75.0%	25.7%	41.7%	47.6%	50.0%	45.4%
In-hospital procedures						
PCI without stent	0.0%	4.6%	0.0%	6.1%	0.0%	5.3%
PCI with bare metal stent	0.0%	8.3%	0.0%	2.5%	20.0%	27.2%
PCI with drug eluting stent	0.0%	14.7%	0.0%	6.8%	60.0%	58.4%
CABG	0.0%	11.0%	8.3%	39.0%	0.0%	2.9%
Number of vessels with CAD						
None	50.0%	33.0%	33.3%	22.4%	10.0%	3.9%
Single	12.5%	25.7%	16.7%	18.7%	50.0%	42.0%
Multiple	37.5%	41.3%	50.0%	58.9%	40.0%	54.1%
Hospital length of stay (mean ± std)	10.8 ± 7.14	4.8 ± 6.46	3.0 ± 1.91	7.3 ± 7.86	2.2 ± 1.40	3.4 ± 4.54

Note: AC: anticoagulant; CVD: cardiovascular disease; MI: myocardial infarction; COPD: chronic obstructive pulmonary disease; CVA: cerebral vascular accident; ICH: intracranial hemorrhage; VTE: vascular thrombus event; AF: atrial fibrillation; STEMI: ST elevated myocardial infarction; PCI: percutaneous coronary intervention; CABG: coronary artery bypass graph; CAD: coronary artery disease.

**Table 3:** Patient characteristics by postdischarge AT therapy.

	No antiplatelet		Single antiplatelet		Dual antiplatelet	
	No AC	AC	No AC	AC	No AC	AC
	*n* = 103	*n* = 23	*n* = 898	*n* = 179	*n* = 3818	*n* = 273
Age (mean ± std)	64.9 ± 15.18	67.6 ± 12.76	64.3 ± 12.29	67.7 ± 11.98	63.8 ± 12.26	67.8 ± 12.50
Male	61.2%	69.6%	70.7%	66.5%	73.7%	76.6%
Caucasian	94.2%	91.3%	87.9%	90.5%	89.5%	92.3%
Body Mass Index (mean ± std)	28.2 ± 5.97	30.6 ± 8.86	29.9 ± 7.27	30.2 ± 18.39	29.7 ± 6.81	30.6 ± 13.87
Family history of early CVD	31.1%	17.4%	43.7%	37.4%	45.5%	37.0%
Smoking history	8.7%	26.1%	16.0%	12.8%	16.3%	17.2%
Prior MI	19.4%	8.7%	15.4%	17.3%	16.7%	17.9%
Comorbidities						
COPD	14.6%	13.0%	6.7%	10.1%	7.1%	12.1%
CVA	6.8%	8.7%	3.9%	10.1%	3.5%	7.7%
Diabetes	32.0%	17.4%	29.3%	25.7%	24.8%	28.6%
Heart failure	38.8%	56.5%	29.4%	49.2%	20.1%	42.9%
Hypercoagulability	1.0%	4.3%	0.0%	1.1%	0.1%	0.7%
Hyperlipidemia	64.1%	56.5%	64.7%	57.5%	65.6%	60.1%
Hypertension	66.0%	52.2%	65.9%	59.8%	65.6%	65.2%
Major bleeding or ICH	5.8%	0.0%	2.7%	2.8%	1.8%	3.7%
Renal failure	17.5%	13.0%	7.9%	10.6%	5.8%	8.4%
Valve disease	14.6%	30.4%	8.2%	19.6%	7.9%	16.1%
VTE	7.8%	43.5%	5.1%	30.7%	3.9%	25.3%
AF						
No AF	71.8%	26.1%	78.8%	37.4%	88.6%	49.1%
Prior but not current	12.6%	17.4%	3.9%	5.6%	3.7%	7.0%
Prior and current	6.8%	30.4%	3.0%	22.4%	2.8%	23.1%
New onset	8.7%	26.1%	14.3%	34.6%	4.9%	20.9%
Charlson Comorbidity Index (mean ± std)	7.6 ± 4.53	8.1 ± 4.04	6.4 ± 3.93	8.2 ± 3.86	5.7 ± 3.76	7.6 ± 3.87
Mechanical value	0.0%	0.0%	0.1%	1.7%	0.2%	0.4%
Presentation						
Unstable angina	70.9%	56.5%	49.1%	35.2%	39.8%	27.1%
STEMI	3.9%	0.0%	6.0%	8.9%	14.9%	22.7%
Non-STEMI	25.2%	43.5%	44.9%	55.9%	45.3%	50.2%
In-hospital procedures						
PCI without stent	3.9%	8.7%	6.1%	7.8%	5.1%	6.2%
PCI with bare metal stent	1.9%	0.0%	1.3%	1.7%	27.4%	37.0%
PCI with drug eluting stent	1.0%	4.3%	2.7%	2.8%	61.5%	50.2%
CABG	2.9%	13.0%	45.7%	32.4%	1.3%	0.7%
Number of vessels with CAD						
None	30.1%	43.5%	18.8%	29.1%	3.9%	7.0%
Single	15.5%	13.0%	14.8%	22.3%	44.1%	35.2%
Multiple	54.4%	43.5%	66.4%	48.6%	52.0%	57.9%
Hospital length of stay (mean ± std)	9.2 ± 8.32	11.1 ± 11.11	7.2 ± 6.93	11.4 ± 11.19	3.0 ± 3.50	6.1 ± 8.62
Hospital discharged home	81.6%	69.6%	83.4%	68.7%	94.6%	83.5%

Note: AC: anticoagulant; CVD: cardiovascular disease; MI: myocardial infarction; COPD: chronic obstructive pulmonary disease; CVA: cerebral vascular accident; ICH: intracranial hemorrhage; VTE: vascular thrombus event; AF: atrial fibrillation; STEMI: ST elevated myocardial infarction; PCI: percutaneous coronary intervention; CABG: coronary artery bypass graph; CAD: coronary artery disease.

**Table 4:** Postdischarge AT therapyfor specific procedures and AC indications (i.e., AF with CHADS_2_ score ≥2 or VTE during hospitalization). The bolded cells are the suggested postdischarge AT therapy based on one or more guidelines.

Procedures	AC indication	Total *N*	No antiplatelet		Single antiplatelet		Dual antiplatelet	
			No AC	AC	No AC	AC	No AC	AC
Stent	No AC indication	3097	0.0%	0.0%	1.0%	0.1%	**95.1%**	3.8%
	AF with CHADS_2_ ≥2	298	0.7%	0.3%	0.7%	0.7%	70.8%	**26.8%**
	VTE during hospitalization	37	0.0%	0.0%	5.4%	5.4%	43.2%	**46.0%**

CABG	No AC indication	406	0.5%	0.3%	**85.0%**	6.7%	7.1%	0.5%
	AF with CHADS_2_ ≥2	103	1.0%	1.9%	58.3%	**21.4%**	17.5%	0.0%
	VTE during hospitalization	16	0.0%	0.0%	31.3%	**56.3%**	12.5%	0.0%

PCI No Stent	No AC indication	250	1.2%	0.0%	18.0%	2.8%	**74.8%**	3.2%
	AF with CHADS_2_ ≥2	31	3.2%	6.5%	29.0%	12.9%	25.8%	**22.6%**
	VTE during hospitalization	6	0.0%	0.0%	16.7%	50.0%	0.0%	**33.3%**

Note: AT: antithrombotic treatment; AC: anticoagulant; VTE: vascular thrombus event; AF: atrial fibrillation; PCI: percutaneous coronary intervention; CABG: coronary artery bypass graph.
