# Supplementary material for: Antithrombotic Therapy in Patients with Acute Coronary Syndrome in the Intermountain Heart Collaborative Study
Source: Cardiol Res Pract. 2015 Jan 8;2015:270508. doi: 10.1155/2015/270508 (PMC4302371; doi:10.1155/2015/270508)

Supplementary Table 1: ICD-9 codes for comorbidities

| **Comorbidity** | **ICD-9 codes** |
| --- | --- |
| COPD | 491, 495, 492, 494, 496 |
| CVA | 433.x1, 434.x1 |
| Diabetes | 250 |
| Heart Failure | 428 |
| Hypercoagulbility | 289.81 |
| Major Bleed | 632.81, 376.32, 379.23, 431, 432,568.81, 719.1[0-9], 800.[2,3,7,8] 801.[2,3,7,8], 803.[2,3,7,8], 804.[2,3,7,8], 851, 852, 853 |
| ICH | 431, 432, 800.[2,3,7,8] 801.[2,3,7,8], 803.[2,3,7,8], 804.[2,3,7,8],852, 853 |
| RenalFailure | 586, 584, 585.[5,6] |
| ValveDisease | 391, 394, 395, 396, 397, 398, 392.0, 424.0, 424.1, 424.2, 424.3 |
| VTE | 415.[1,11,19], 451.[1,11,19,81], 453.[2 ,40,41,42,50,51,52,6,71,72,73,74,75,76,77,79,8,81,82,83,84,85,86,87,89,9], 671.[30,31,33,40,42,44], 673.[2,20,21,22,24,24] |

Supplementary Table 2: Medications for In-hospital and Post Discharge

| **AT Category** |  | In-Hospital | | Post-Discharge | |
| --- | --- | --- | --- | --- | --- |
|  | **Medications** | **N** | **%** | **N** | **%** |
| **AC only** |  |  |  |  |  |
|  | Other Anti-Coagulant | 93 | 85.3% | 0 | 0.0% |
|  | Warfarin, & Other Anti-Coagulant | 11 | 10.1% | 0 | 0.0% |
|  | Warfarin | 5 | 4.6% | 23 | 100.0% |
| **SAP and no AC** |  |  |  |  |  |
|  | Aspirin | 12 | 100.0% | 856 | 95.3% |
|  | Clopidogrel | 0 | 0.0% | 40 | 4.5% |
|  | Other Anti-Platelet | 0 | 0.0% | 2 | 0.2% |
| **SAP and AC** |  |  |  |  |  |
|  | Aspirin and Other Anti-Coagulant | 748 | 74.8% | 0 | 0.0% |
|  | Aspirin, Warfarin, & Other Anti-Coagulant | 178 | 17.8% | 0 | 0.0% |
|  | Clopidegrel & Other Anti-Coagulant | 49 | 4.9% | 0 | 0.0% |
|  | Aspirin & Warfarin | 17 | 1.7% | 173 | 96.6% |
|  | Clopidegrel, Warfarin, & Other Anti-Coagulant | 6 | 0.6% | 0 | 0.0% |
|  | Other Anti-Platelet & Other Anti-Coagulant | 2 | 0.2% | 0 | 0.0% |
|  | Clopidegrel & Warfarin | 0 | 0.0% | 5 | 2.8% |
|  | Other Anti-Platelet & Warfarin | 0 | 0.0% | 1 | 0.6% |
| **DAP and no AC** |  |  |  |  |  |
|  | Aspirin, Clopidegrel, & Other Anti-Platelet | 5 | 50.0% | 21 | 0.6% |
|  | Aspirin & Other Anti-Platelet | 3 | 30.0% | 11 | 0.3% |
|  | Aspirin & Clopidegrel | 2 | 20.0% | 3779 | 99.0% |
|  | Clopidegrel & Other Anti-Platelet | 0 | 0.0% | 7 | 0.2% |
| **DAP and AC** |  |  |  |  |  |
|  | Aspirin, Clopidegrel, & Other Anti-Coagulant | 3534 | 85.1% | 0 | 0.0% |
|  | Aspirin, Clopidegrel, Warfarin, & Other Anti-Coagulant | 407 | 9.8% | 0 | 0.0% |
|  | Aspirin, Clopidegrel, Other Anti-Platelet, & Other Anti-Coagulant | 139 | 3.3% | 0 | 0.0% |
|  | Aspirin, Other Anti-Platelet, & Other Anti-Coagulant | 49 | 1.2% | 0 | 0.0% |
|  | Aspirin,Other Anti-Platelet, Warfarin, & Other Anti-Coagulant | 8 | 0.2% | 0 | 0.0% |
|  | Aspirin, Clopidegrel, Other Anti-Platelet, Warfarin, & Other Anti-Coagulant | 7 | 0.2% | 0 | 0.0% |
|  | Clopidegrel, Other Anti-Platelet, & Other Anti-Coagulant | 5 | 0.1% | 0 | 0.0% |
|  | Aspirin, Clopidegrel, & Warfarin | 5 | 0.1% | 267 | 97.8% |
|  | Clopidegrel, Other Anti-Platelet, Warfarin, & Other Anti-Coagulant | 1 | 0.0% | 0 | 0.0% |
|  | Aspirin, Clopidegrel, Other Anti-Platelet, & Warfarin | 0 | 0.0% | 4 | 1.5% |
|  | Aspirin, Other Anti-Platelet, & Warfarin | 0 | 0.0% | 2 | 0.7% |
|  | Clopidegrel, Other Anti-Platelet, & Warfarin | 0 | 0.0% | 0 | 0.0% |

Supplementary Figure 1: Univariate and Multivariable Logistic Regression for In-Hospital SAP vs DAP Use


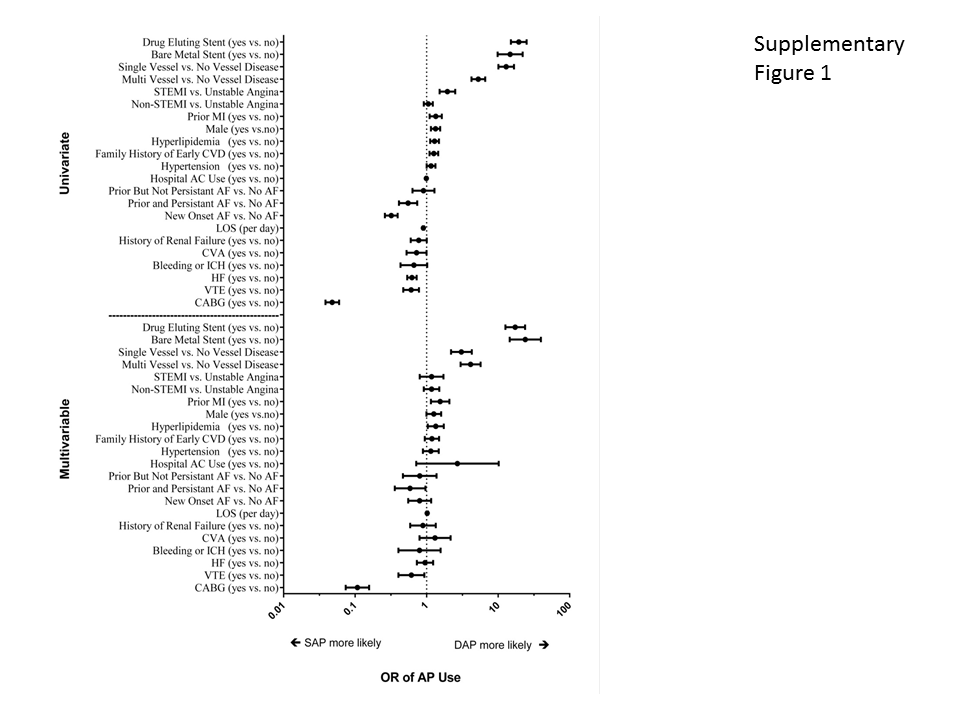

Supplement: Supplementary file 1 — The supplementary materials include two tables and one figure. The first table contains the ICD-9 codes that were used to define each of the comorbidities. The second table contains the actual medications that make up each category of the single (SAP) or dual (DAP) antiplatelet and anticoagulants (AC). The figure contains the univariate and multivariable logistic regression for odd ratios for factors associated with in-hospital SAP vs DAP use. [file 270508.f1.docx]
